# Impact of Diagnosis and Treatment of Chronic Endometritis on Outcomes Before Starting Assisted Reproductive Technology: A Retrospective Study

**DOI:** 10.1007/s43032-024-01633-5

**Published:** 2024-07-09

**Authors:** Kazuyoshi Sakai, Isao Takehara, Hiromu Kaneko, Fumihiro Nakamura, Nanako Nakai, Kyoko Takahashi, Jun Matsukawa, Koki Matsuo, Satoru Nagase

**Affiliations:** Department of Obstetrics & gynecology, Yamagata University School of Medicine, 2-2-2 Iida-Nishi, Yamagata-shi, Yamagata 990-9585 Japan

**Keywords:** Chronic endometritis, CE, CD138, ART, Antimicrobial agents

## Abstract

This study aimed to investigate the effect of diagnosis and treatment of chronic endometritis (CE) on the outcome of assisted reproductive technology (ART) with or without repeated implantation failure (RIF). This retrospective analysis included patients who underwent pathological examination for diagnosis of CE at Yamagata University Hospital. The examination was performed for all patients planned for ART with or without RIF. Patients who were examined within 6 months of the first oocyte retrieval or embryo transfer were included. We counted the number of CD138-positive cells within the endometrial stroma in patients’ specimens and analyzed the patients’ clinical information. Clinical rates of pregnancy and implantation were determined. A total of 80 women met the inclusion criteria: 13 CE-negative patients (17.3%) and 67 CE-positive patients (83.7%). A significant decrease was noted in the CD138-positive cell count between the first biopsy and second biopsy after CE treatment (*p* < 0.001). In addition, no significant differences were noted in ongoing pregnancy rates between the CE-negative patients and those who underwent CE treatment. The CD138-positive cell counts at first biopsy tended to be lower in each pregnancy group than in the non-pregnancy group. For patients planned to undergo ART, examination for diagnosis of CE with or without RIF could be considered. Pathological CD138-positive cell counts were considered useful for CE diagnosis and treatment decision-making. The study findings suggest the efficacy of antimicrobial agents in CE treatment, contributing to improved pregnancy outcomes.

## Introduction

Chronic endometritis (CE) is characterized by the persistence of bacteria or other antigens in the uterus after menstrual periods, eliciting an immunological response in the endometrium [1]. CE has been reported to be one of the causes of repeated implantation failure (RIF) in assisted reproductive technology (ART) [2], prompting widespread diagnostic and therapeutic efforts across various medical facilities.

Previous studies have reported that CE is diagnosed in approximately 14%–67.5% of RIF patients [3–8], and the efficacy of oral antibiotic treatment has been documented [5, 9, 10]. Furthermore, successful resolution of CE in RIF patients has been associated with improved fertility outcomes [11]. Despite these advancements, practical challenges persist with two major problems. First, the diagnosis of CE often occurs after multiple embryo transfer attempts for the following reason. Currently, examination for diagnosis of CE is indicated only for patients with RIF. RIF is typically diagnosed in ART settings when four or more good embryos and three or more transfers fail to result in pregnancy [2]. In other words, patients with CE are forced to undergo multiple embryo transfers before treatment of CE. There could be a substantial number of CE at the time of starting ART because CE has been reported in approximately 2.8%–56.8% of all infertile patients [12–14]. Therefore, we believe that early examination of CE, possibly conducted not for the patients with RIF but before the first embryo transfer like as screening test, may improve pregnancy outcomes by facilitating timely CE treatment. Second, methods of examination and diagnostic criteria for CE have not been established; therefore, indications for treatment vary across medical facilities. Currently, the main diagnostic method involves quantification of CD138-positive cells in endometrial tissue; however, common criteria with a universally accepted threshold for CE-positive cell count have not been defined [15]. It can be useful to create an index of diagnosis based on the count of CD138 cells, which is an objective assessment, rather than hysteroscopy, which relies on subjective assessment.

Therefore, this study aimed to examine the effect of the diagnosis and treatment of CE on the outcome of ART and report a retrospective review of our experience in examination and treatment of CE in ART patients, regardless of the diagnosis of RIF, at our institution.

## Materials and Methods

This study was approved by the Ethics Committee of Yamagata University Faculty of Medicine (approval number: 202061).

Patients who underwent pathological examination for diagnosis of CE between January 2019 and December 2020 at Yamagata University Hospital were included in the study. During this period, if a patient undergoing ART requested for examination of CE, it was performed regardless of diagnosis of RIF. Therefore, the study cohort included both cases before and after the start of ART. To reduce the effect of timing of the examination, only patients who were examined within 6 months of the first oocyte retrieval or embryo transfer (ET) were included. Patient information including age, gravidity, parity, serum anti-mullerian hormone (AMH) level, antral follicle count (AFC), number of cycles of artificial insemination or ET performed prior to diagnosis of CE, and underlying diseases [uterine myoma, endometriosis, polycystic ovarian syndrome (PCOS), endometrial polyps] that may have contributed to infertility was extracted from their medical records. Patients with factors of infertility other than CE were excluded: those who were unable to obtain embryos due to poor ovarian reserve or fertilization failure, those who were unable to obtain an endometrial tissue biopsy, those who became pregnant by methods other than embryo transfer, and those who did not visit the hospital after pathological examination.

### Diagnosis of CE

We performed biopsy of endometrial tissue during the follicular phase within 6 months of the first oocyte retrieval or embryo transfer. First, hysteroscopic observation of intrauterine tissue was performed, and after removing sufficient saline solution from the uterine cavity, endometrial biopsy was conducted using endosuction*®* (HAKKO CO, Japan). The endometrium was pathologically fixed, and the specimen was stained with CD138. Pathological diagnosis of CE was performed through counting the number of CD138-positive cells within the endometrial stroma in the specimen. Immunostaining was performed, and the pathological specimens were evaluated by pathologists. The CD138 cell count was defined as the total number of positive cells in 10 high power fields (HPFs). A CE-positive specimen was defined as having at least one CD138-positive cell count in 10 HPFs, whereas a CE-negative specimen was defined as having a zero CD138-positive cell count in 10 HPFs. The successful therapy of CE was defined as zero CD138-positive cell count on the second biopsy after the antibiotic medication, whereas residual CE was defined as 1 or more CD138-positive cell count after antibiotic medication. Intrauterine findings using hysteroscopes were considered only for reference findings and were not considered as treatment criteria.

### Treatment of CE

**Fig. 1:**
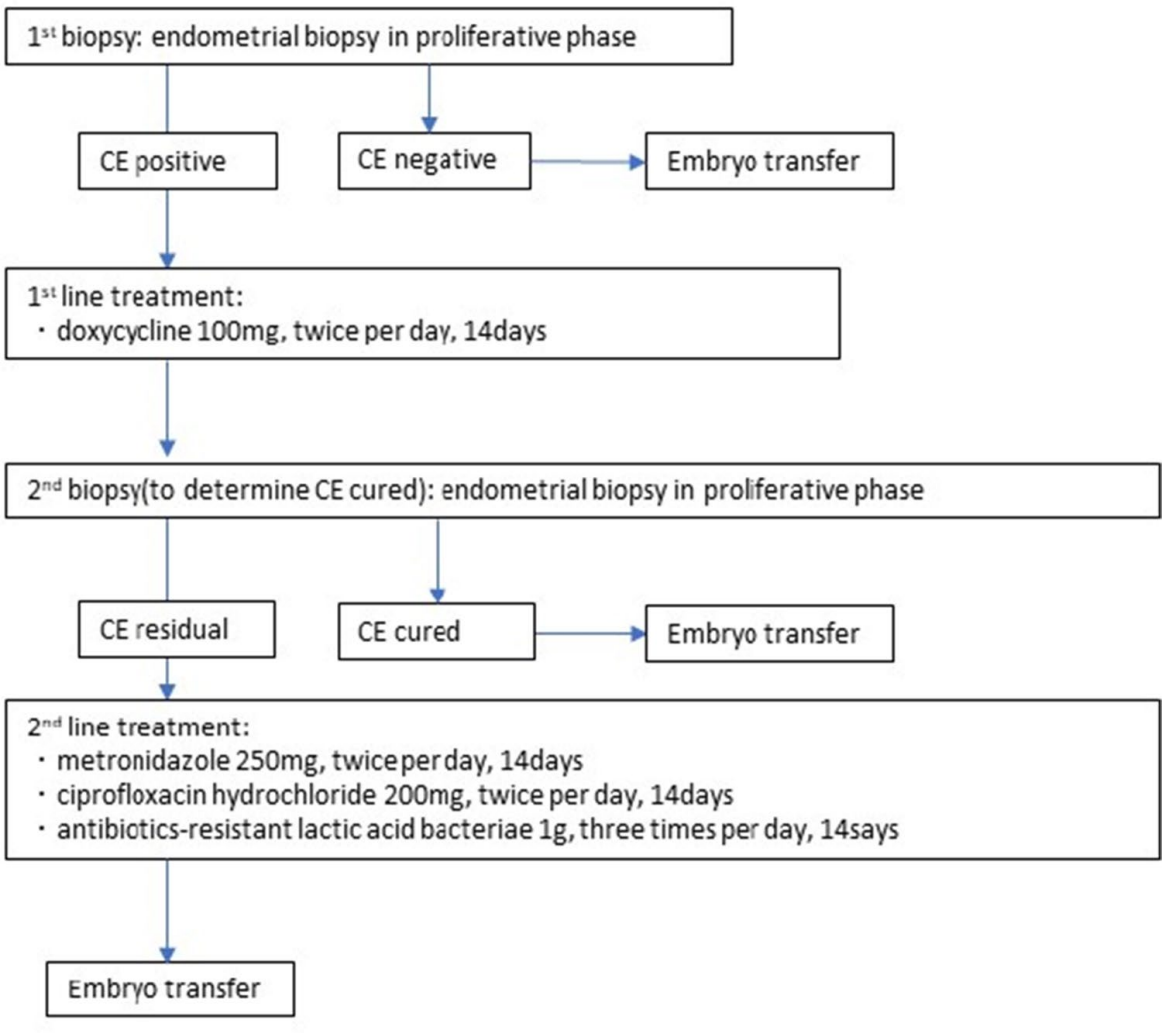
Pathological examination and oral antibiotic treatment protocol for CE at our hospital. CE: chronic endometritis

The treatment protocol for CE at our hospital between January 2019 and December 2020 is depicted in Fig. 1. For antimicrobial selection, we referred to the previous reports [5, 16] because 70–90% of plasma cells were reported to have been reduced. The first-line treatment comprised 100 mg of oral doxycycline, administered twice per day, for 14 days. The duration of dosing in the menstrual cycle were not specifically standardized. To determine the efficacy of CE treatment, the endometrial tissue was biopsied and immunostained again (second biopsy) in the proliferative phase immediately after the end of oral administration of the above-mentioned medication. The group that had residual CE after the first dose was treated with second-line drugs. The second-line treatment comprised oral metronidazole (250 mg, twice per day), ciprofloxacin hydrochloride (200 mg, twice per day), and antibiotic-resistant lactic acid bacteria (1 g, three times per day), administered for 14 days. After the second-line treatment was administered, the treatment of CE was terminated, and the plan was to perform ET in all patients.

### Outcomes

Outcomes were defined as positive proof for pregnancy test (serum human chorionic gonadotropin: hCG levels rising above 20 mIU/mL around day 10 after embryo transfer) and ongoing pregnancy or live birth. An ongoing pregnancy was defined as a pregnancy that continued for more than 12 weeks.

### Statistical Analysis

R software was used for statistical analysis. One-way analysis of variance (ANOVA), Fisher's exact probability, Mann-Whitney U, and Wilcoxon signed rank sum tests were used. A *P*-value < 0.05 was considered statistically significant.

## Results

In this study, 110 patients underwent pathological examination of CE between January 2019 and December 2020; of these, 80 were included in the study. Overall, 30 patients were excluded as follows: 14 patients could not undergo endometrial biopsy (Timing of the test was not right, or there was insufficient specimen, etc.), 5 patients got pregnant without embryo transfer (natural pregnancy), and 11 patients could not undergo embryo transfer after the biopsy (Abandoned treatment, transferred to another hospital, etc.). At the first biopsy, there were 13 CE-negative patients (16.3%, defined as the “CE-negative group”) and 67 CE-positive patients (83.7%). At the second biopsy, wherein another biopsy was performed after the first-line treatment for the 67 CE-positive patients, CD138 cells disappeared in 21 patients (defined as “CE first-line group”), and the remaining 46 patients were subjected to second-line treatment (defined as “CE second-line group”). No significant differences were noted between these three groups, including history of pregnancy and infertility treatment (Table 1). A slightly higher number of patients with uterine myoma and endometriosis was observed in the CE-negative group; however, it was considered that the results would not be affected because of the small number of patients.

**Table 1:** Characteristics of study participants

	CE negative (n = 13)	CE first-line group (n = 21)	CE second-line group (n = 46)	*p*
Age	36.4 (32–41)	36.08 (29–42)	37.5 (28–44)	0.346
Multigravida	7 (53.8%)	8 (38.1%)	20 (43.4%)	0.675
Multipara	4(%)	5 (%)	9 (19.5%)	0.726
AMH (ng/mL)	3.28 (1.36–9.75)	3.74 (0.5–10.04)	4.68 (0.13–13.9)	0.227
AFC	8.8 (5–13)	11.7 (2–22)	12.1 (6–21)	0.113
AIH cycles	3.6 (0–9)	3.0(0–11)	2.5 (0–8)	0.432
ET cycles	1.5 (0–6)	1.9 (0–9)	1.5 (0–10)	0.803
Myoma	4 (30.8%)	2 (9.5%)	3 (6.5%)	0.0483
Endometriosis	5 (38.5%)	1(%)	3 (6.5%)	0.00246
PCOS	0 (0%)	2 (9.5%)	2 (%)	0.453
Endometrial polyp	1 (%)	1 (%)	2 (1%)	0.636

CE: chronic endometritis; AIH cycles: number of the cycles of artificial insemination with husband performed prior to CE testing; ET cycles: number of cycles of embryo transfer performed prior to CE testing; AMH: serum anti-mullerian hormone level; AFC: antral follicle count; PCOS: polycystic ovarian syndrome; CE first-line group: patients cured of CE after first-line treatment; CE second-line group: patients treated after second-line treatment

The main outcome of the study is shown in Table 2. We compared outcomes in the CE-negative, CE first-line, and CE second-line groups. No significant differences were noted in ongoing pregnancy/live birth rates between these three groups (CE-negative, CE first-line, and CE second-line groups: 46.1%, 47.6%, and 39.1%, respectively, *p* = 0.78); however, the CE second-line group (54.3%) showed significantly lower pregnancy rates than did the CE-negative group (84.6%, *p* = 0.01) or CE first-line group (76.1%, *p* = 0.02). The pregnancy rates were not significantly different between the CE-negative and CE first-line groups (*p* = 0.56).

**Table 2:** Main outcome

	CE negative (n = 13)	CE first-line group (n = 21)	CE second-line group (n = 46)
pregnancy*	11	16	25
pregnancy rate (%)	84.6% ^a^	76.1% ^a^	54.3% ^b^
ongoing pregnancy or live birth	6	10	18
ongoing pregnancy or live birth rate (%)	46.1%	47.6%	39.1%
			*p* = 0.782

Values with different superscript letters within the same column are significantly different; *P* < 0.05

Pregnancy: positive proof for pregnancy test (serum human chorionic gonadotropin: hCG level rising above 20 mIU/mL around day 10 after embryo transfer)

**Fig. 2:**
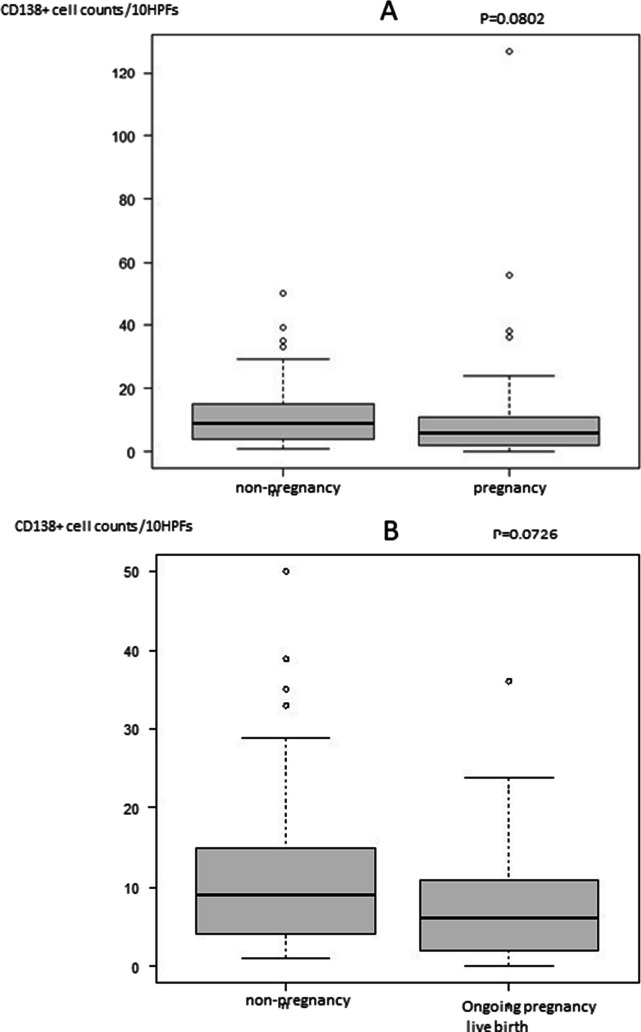
**A**: Comparison of CD138 positive cell counts between non-pregnancy and pregnancy groups at first biopsy. **B**: Comparison of CD138 positive cell counts between non-pregnancy and ongoing pregnancy or live birth groups at first biopsy

**Fig. 3:**
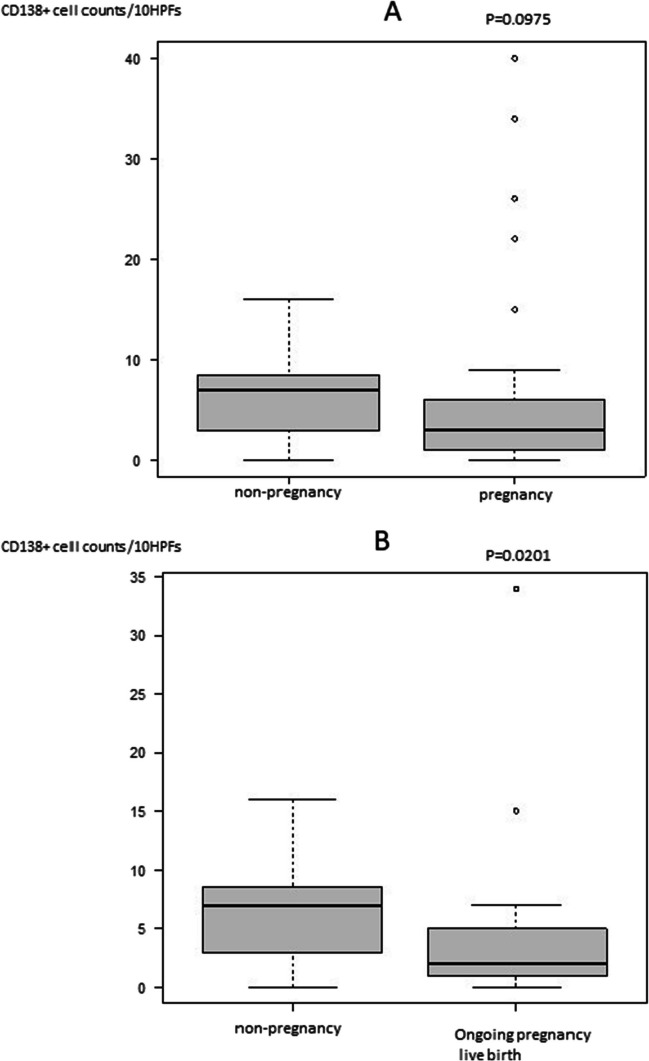
**A**: Comparison of CD138 positive cell counts between non-pregnancy and pregnancy groups at second biopsy performed to diagnose whether CE has been cured. **B**: Comparison of CD138 positive cell counts between non-pregnancy and ongoing pregnancy or live birth groups at second biopsy. CE: chronic endometritis

A significant decrease was noted in the number of CD138-positive cells between the first and secondary biopsies (*p* < 0.001). Further, we evaluated whether the number of CD138-positive cells affected pregnancy outcome regardless of treatment. The number of CD138-positive cells at the first biopsy tended to be lower in the pregnancy groups than in the non-pregnancy group: 9 (4–15) cells /10 HPFs, 6 (4–9) cells /10 HPFs, *p* = 0.080 (Fig. 2A). Further, no significant difference was noted in the non-pregnancy group and ongoing pregnancy/live birth group: 6 (2.25–10.25) cells /10 HPFs, *p* = 0.073 (Fig. 2B). In addition, the number of CD138-positive cells at the second biopsy tended to be lower in the pregnancy group than in the non-pregnancy group: non-pregnancy and pregnancy group, 7 (3–8.5) cells /10 HPFs and 3 (1–6) cells /10 HPFs, respectively, *p* = 0.098. Meanwhile, the number of CD138-positive cells at the second biopsy was significantly lower in the ongoing pregnancy or live birth group than that in the non-pregnancy group (non-pregnancy, ongoing pregnancy or live birth: 7 (3–8.5) cells /10 HPFs, 2 (1–5) cells /10 HPFs, *p* = 0.020 (Fig. 3).

## Discussion

This is the first study investigating the association between CE and ART outcomes regardless of the existence of RIF. Unexpectedly, we found that many patients undergoing ART before the diagnosis of RIF already had CE. For these patients, antibiotic treatment of CE certainly reduced the number of CD138-positive cells, serving as a possible predictor of subsequent fertility outcomes. These factors suggest that the diagnosis and treatment of CE may be considered not only for patients diagnosed with RIF but also for all patients planning to undergo ART. In addition, our study suggests the efficacy of evaluating CD138-positive cell count in the diagnosis and treatment of CE. We believe that the indications for the diagnosis and treatment of CE could be expanded beyond the currently existing ones.

CE is a chronic inflammation condition of the endometrium caused by various factors. Previous reports have included history of intrauterine device (IUD) insertion [17, 18], multiple pregnancies or abnormal uterine bleeding [17], and bacterial vaginosis, endometrial polyps, or endometriosis [19–22] as causes.

In this study, we evaluated whether background characteristics of patients, including history of intrauterine manipulation (artificial insemination or ET), affected the occurrence of CE; however, we could not find any specific correlation. Although significant differences were noted for myoma and endometriosis, these factors need not be necessarily considered, as the number of patients in this study were very small.

Generally, diagnosis and treatment of CE should be performed on patients with RIF; however, we consider that all patients who are planning to undergo ART should be indicated. Currently, CE has been reported in approximately 2.8%–56.8% of infertile patients [12–14], approximately 14%–67.5% of RIF patients [3–8], and approximately 9.3%–67.6% of patients with repeated miscarriages [23, 24]. This variation among reports might be attributed to the different examination methods and diagnostic criteria. In this study, we defined CE positivity as “CD138-positive cells ≥ 1/10 HPFs,” with reference to a previous study [25], and the positivity rate was as high as 83.7% for patients planning to undergo ART with or without RIF. Although a relatively strict definition, such as the one mentioned above, may have influenced this high rate, it was suggested that a significant proportion of patients planning ART could already have CE. Meanwhile, the number of CD138-positive cells was significantly decreased when antimicrobial agents were administered to patients with CE in this study. A high number of CD138-positive cells in the endometrium is thought to have a negative effect on the pregnancy and ongoing pregnancy rates in ART [26–28]. Hence, decreasing CD138 positive cells with antimicrobial therapy for such CE patients may be beneficial for pregnancy. In practice, no significant differences were noted in the ongoing pregnancy rates between the CE-negative group and the groups after CE treatment. It is possible that CE treatment reduced the number of CD138-positive cells, resulting in improved and comparable pregnancy outcomes in the CE-positive patients. However, the low pregnancy rate in the CE second-line group suggests that the treatment effect may not be sufficient in severe cases; therefore, early diagnosis of CE and first-line treatment may be beneficial before it becomes severe. In the ongoing pregnancy or live birth group, CD138-positive cell counts were significantly lower at the second biopsy (Fig. 3), suggesting that the reduction in the number of CD138 cells caused by first-line antibiotic treatment could be important. Taken together, it may be useful to consider patients with diagnosis of CE and antibiotic treatment before the planned ART stage.

Our results also suggest the efficacy of evaluating the number of CD138-positive cells in the endometrium in the diagnosis of CE. To the best of our knowledge, no study has reported on pathological evaluation before and after antimicrobial therapy. Currently, CE is diagnosed through hysteroscopy, pathological examination, bacteriological testing, or a combination of two of these methods, with many medical facilities generally using both hysteroscopy and pathological examinations, given their simplicity [8, 16, 21]. During hysteroscopy, “strawberry” aspect, focal hyperemia, hemorrhagic spots, diffuse micropolyps, and stromal edema have been reported as findings that predict CE [29]. Using hysteroscopy, the diagnosis can be made immediately upon examination. However, even in cases where hysteroscopy shows no abnormal findings, pathological examinations often reveal CE. A review by *Fani et al*. [8] stated that hysteroscopy was not suitable as a first choice for CE diagnosis because of the high risk of bias. Similarly, in our study, the findings of hysteroscopy were often viewed differently by each physician. The positive predictive value of CE through hysteroscopy to pathological examination was 0.886 (95% confidence interval 0.754–0.962), whereas the negative predictive value was only 0.222 (95% confidence interval 0.101–0.392) (data not shown). Therefore, it was considered that hysteroscopy should be in the category of adjunctive diagnosis, as it could not provide reproducible results. Meanwhile, CD138 immunostaining specific for plasma cells has been commonly used for pathological diagnosis of CE. A higher number of CD138-positive cells was associated with reduced subsequent fertility outcomes, and the number of CD138-positive cells was considered to be able to assess the disease status of CE [13, 14, 23]. Although pathological examination took longer than hysteroscopy, it had the advantage of greater objectivity and less bias [8]. Our study also suggested that the number of CD138-positive cells could be a predictor of pregnancy outcome through evaluating CD138-positive cells before and after treatment.

The standard treatment for CE comprises administration of antimicrobial agents, but no general regimen has been established to date. *Kitaya et al.* reported that 92.3% of patients with CE improved upon treatment with doxycycline (200 mg/day for 14 days), while the remaining patients received ciprofloxacin (400 mg/day for 14 days) and metronidazole (500 mg/day for 14 days), and reported an overall response rate of 99.1% [16]. We referred to this as the treatment protocol at our facility. In other studies, a combination of doxycycline, ciprofloxacin, and metronidazole [5] or ofloxacin and metronidazole [9] has been reported to be effective for the treatment of CE. *Cicinelli et al*. treated CE with a systematic antibiotic regimen according to the microbial profile of the endometrium [10]. As described above, various studies have reported on antimicrobial therapy for CE; however, previous reports have only determined whether CE was cured and have not discussed the reduction in the number of CD138-positive cells after antibiotic treatment. In this study, we found for the first time that the number of CD138-positive cells was significantly reduced after antimicrobial therapy, which can be useful as an indicator for evaluating the efficacy of antimicrobial treatment.

This study has several limitations. We could not evaluate the number of CD138-positive cells after second-line antimicrobial treatment pathologically. Therefore, the CE of some patients in the treatment group may not yet be cured. In such cases, further treatment of CE may need to be considered. Second, because this study was a retrospective study, and all patients who had CE were treated, direct comparisons between untreated and treated cases were not possible. Further studies with prospective designs are needed to evaluate the accurate effect of CE treatment. However, the usefulness of the pathological examination of CE and the therapeutic effect were at least demonstrated through our study.

CE was found to be more common than that expected in patients planning for ART. The findings suggest that antimicrobial therapy in these patients may have benefited pregnancy outcomes. Further, the diagnosis and treatment of CE should be considered not only for patients with RIF but also for all patients planning to undergo ART like as screening test. In addition, the evaluation of CD138-positive cell count was found to be objective as well as useful as an indicator of treatment. Therefore, pathological examination could be the preferred assessment for diagnosis of CE. Furthermore, many CE-positive patients are said to have an abnormal pattern in endometrial receptivity analysis (ERA) [30]. Taken together, early detection of CE is expected to be a novel key investigation in future ART treatment strategies. Further research is needed on these relationships, including the availability and efficacy of treatment of CE and endometrial receptivity. We plan to conduct these additional studies of early CE treatment and other adjunctive therapies.

In conclusion, for all patients planning to undergo ART, examination for diagnosis of CE should be considered, regardless of existence of RIF, and pathological CD138-positive cell counts could be useful as a method for diagnosis of CE and treatment decision-making. In addition, antimicrobial agents could be useful in the treatment of CE, contributing to better pregnancy outcomes.

## Data Availability

The data are available from the corresponding author, I.T. upon reasonable request.
